# Molecular effect of an OPTN common variant associated to Paget's disease of bone

**DOI:** 10.1371/journal.pone.0197543

**Published:** 2018-05-21

**Authors:** Iris A. L. Silva, Natércia Conceição, Édith Gagnon, Jacques P. Brown, M. Leonor Cancela, Laëtitia Michou

**Affiliations:** 1 Department of Biomedical Sciences and Medicine, and Algarve Biomedical Center, University of Algarve, Faro, Portugal; 2 Centre of Marine Sciences (CCMAR), University of Algarve, Faro, Portugal; 3 Research centre of the CHU de Québec-Université Laval, Québec City, QC, Canada; 4 Division of Rheumatology, Department of Medicine, Université Laval and Department of Rheumatology, CHU de Québec-Université Laval, Québec City, QC, Canada; Charles P. Darby Children's Research Institute, UNITED STATES

## Abstract

Paget’s disease of bone (PDB) is a chronic bone disorder and although genetic factors appear to play an important role in its pathogenesis, to date PDB causing mutations were identified only in the *Sequestosome 1 (SQSTM1)* gene at the *PDB3* locus. *PDB6* locus, also previously linked to PDB, contains several candidate genes for metabolic bone diseases. We focused our analysis in the most significantly associated variant with PDB, within the *Optineurin* (*OPTN)* gene, i.e. the common variant rs1561570. Although it was previously shown to be strongly associated with PDB in several populations, its contribution to PDB pathogenesis remains unclear. In this study we have shown that rs1561570 may contribute to PDB since its *T* allele results in the loss of a methylation site in patients’ DNA, leading to higher levels of *OPTN* gene expression and a corresponding increase in protein levels in patients’ osteoclasts. This increase in *OPTN* expression leads to higher levels of NF-κB translocation into the nucleus and increasing expression of its target genes, which may contribute to the overactivity of osteoclasts observed in PDB. We also reported a tendency for a more severe clinical phenotype in the presence of a haplotype containing the rs1561570 *T* allele, which appear to be re-enforced with the presence of the *SQSTM1/P392L* mutation. In conclusion, our work provides novel insight towards understanding the functional effects of this variant, located in *OPTN* intron 7, and its implication in the contribution to PDB pathogenesis.

## Introduction

Paget’s disease of bone (PDB) is a metabolic bone disorder that affects between 1% and 3% of Caucasians over the age of 55 [1–3]. This disease is characterized by focal abnormal bone remodelling, with increased bone resorption and accelerated, excessive and disorganized new bone formation. The pathophysiology of PDB is currently an area of intensive investigation and both genetic and non-genetic factors have been implicated. Fifteen to 40 percent (15–40%) of affected patients have a first-degree relative with PDB and numerous studies have described large families with PDB exhibiting an autosomal dominant mode of inheritance [3–5]. Linkage studies in these families have identified a number of susceptibility loci on chromosomes *6p21* (*PDB1*) [6], *18q21*.*1–22* (*PDB2*) [7], *5q35* (*PDB3*), *5q31* (*PDB4*) [5], *2q36* (*PDB5*) [8], *10p13* (*PDB6*) [9] and *18q23* (*PDB7*) [8]. These regions contain many genes that could be plausible candidates based on their known functions. A genome-wide scan in British families with PDB has shown a linkage to the *10p13* (*PDB6*) locus [9,10] and reanalysis of data from this genome-wide scan confirmed this genetic association [11], namely to the common variant rs1561570 (hg19, chr10:g.13155726 T>C) in the Optineurin (*OPTN*) gene. Our group has also replicated the strong, statistically significant, genetic association of rs1561570 (*p*-value = 5.65×10^−7^) with PDB in the French-Canadian population [12]. *OPTN* gene has been previously linked to glaucoma [13] and neurodegenerative diseases, like Alzheimer’s, Parkinson’s or amyotrophic lateral sclerosis [14]. It was also found to be important for several cellular events such as membrane trafficking and maintenance of the Golgi complex, autophagy and protein turnover through the ubiquitin-proteasome pathway [15–18]. OPTN has been characterized as an autophagy adaptor protein, which regulates selective autophagy of ubiquitin-coated cytosolic proteins, organelles and parasites such as *Salmonella enterica*. This function depends on the phosphorylation of its LC3-interacting motif by TANK-binding kinase 1 (TBK1) [16,18–20]. OPTN can also mediate protein aggregate removal through a ubiquitin-independent mechanism [21] and, in addition, it seems to be part of the TNFα pathway, contributing to mediate the availability of NF-κB in the cell [22,23]. A possible role for OPTN in bone was recently described by Obaid *et al*. (2015) who have shown that mice harbouring a loss-of-function mutation in *Optn* presented an increase in osteoclast activity and in bone turnover, thus indicating that this protein could be acting as a negative regulator of osteoclast differentiation, possibly by inhibiting NF-κB activation [24]. Despite these findings, this animal model did not develop a PDB-like phenotype. Previously, in a different model and in contrast with those results, OPTN was described as being involved in the activation of NF-κB pathway [25]. Indeed, Journo and colleagues (2009) have observed that in human cells, infected with the human T lymphotropic virus type 1, OPTN increased the ubiquitination of Tax1 as well as Tax1-dependent NF-κB signalling, thus promoting NF-κB activation, a result that is in agreement with other studies [26–28]. These discrepancies suggest that the pathologies linked to OPTN cannot be associated exclusively to its loss of function, but more likely to its aberrant expression, resulting from either mutant or gain of OPTN function affecting different pathways in distinct biological contexts, and which are not yet fully understood. Therefore, additional work is required to understand the complexity of the events associated to OPTN function, namely using *ex-vivo* tools such as PDB patients’ samples. In the present work the effect in osteoclastogenesis of the most significantly PDB-associated *OPTN* variant (rs1561570) was assessed using patients’ monocytes as study model. These cells are precursors of osteoclasts and can be induced to fuse in order to originate mature osteoclast cells *in vitro*. The results obtained are expected to contribute to further elucidate the role of *OPTN* in the pathophysiology of a complex chronic bone disease such as PDB.

## Materials and methods

### Study participants

This study was approved by the CHU de Québec-Université Laval Ethics Committee and all participants have signed a consent form before inclusion in the study. We investigated patients with familial form of PDB (one patient per family, to avoid the biais of relatedness in our genetic association analysis), unrelated PDB patients and healthy controls, all from the French-Canadian population. The intra familial cosegregation of the T allele among affected relatives within familial forms was not performed. All patients and healthy donors studied here were non-carrier of the *P392L* mutation within the *SQSTM1* gene (*PDB3* locus). For the PDB osteoclastic phenotype characterization and to assess the potential synergistic effect of rs1561570 with *SQSTM1/P392L* mutation in osteoclastogenesis, we studied a patient heterozygous for rs1561570 (*CT* genotype) and also carrier of the *P392L* mutation in *SQSTM1*. Phenotype assessment, DNA and RNA extraction methods are detailed in the S1 File.

### Bioinformatic analysis

To analyse if rs1561570 was affecting the methylation status at that particular location we used the MethPrimer (http://www.urogene.org/methprimer/), an *in silico* tool used to predict CpG island by examining the CG dinucleotides and to design specific primers to analyse those CpG islands (S1 Table).

### Methylation analysis by bisulfite conversion and sanger sequencing

Sodium bisulfite treatment was performed on 1.5 μg of genomic DNA sample using the EpiMark® Bisulfite Conversion Kit [29] (New England Biolabs Inc., Canada) following the manufacturers’ standard protocol. Further details are provided in the S1 File.

### Cell culture conditions

The U937 (Human monocytes/lymphoma) and U2OS (Human bone osteosarcoma) cell lines were grown in Dulbecco's modified eagle medium (DMEM), supplemented with 10% fetal bovine serum (FBS), 2mM L-glutamine and 1% penicillin/streptomycin (P/S). The T47D (Human breast carcinoma) cell line was grown in RPMI-1640 medium supplemented with 10% FBS, and 1% P/S. The MG63 (Human bone osteosarcoma) cell line was grown in αMEM supplemented with 10% FBS, and 1% P/S. All cell lines were incubated at 37°C in a humidified atmosphere containing 5% CO_2_. The medium, FBS, antibiotics and glutamine were obtained from Invitrogen, Portugal. Cell lines were obtained from American Type Culture Collection (ATCC).

### Demethylating treatment and immunofluorescence

The localization of NF-κB after treatment with 5-Azacitidine (5-Aza, Sigma-Aldrich, Portugal) for 72h in T47D and U937 cell lines was assessed by immunofluorescence. NF-κB localization was also assessed by immunofluorescence in osteoclasts derived from Peripheral Blood Mononuclear Cells (PBMCs) from healthy donors and PDB patients with all studied genotypes. The immunofluorescence protocol is described in the S1 File.

### Preparation of human *in vitro*-differentiated mature osteoclasts

Human mature osteoclasts were differentiated *in vitro* using mononuclear cells from blood of healthy controls and PDB patients. PBMCs were obtained by density gradient centrifugation using Ficoll-Hypaque. The cells were resuspended (3x10^6^ cells/mL) in OPTI-MEM containing 10% FBS. The cell suspension was added to 6-well plates (9x10^6^ cells/well) and to Lab-Tek 8 well-slides (3-6x10^5^ cells/well). After 24h, the cells were washed thoroughly and lymphocyte-free adherent cells were incubated for three weeks with M-CSF (25 ng/ml, Life technologies, Canada) and RANKL (30 ng/ml, Fitzgerald, Canada).

### Osteoclast morphology assessment, TRAP assay and *in vitro* assessment of bone resorption

Mature osteoclast formation was evaluated by quantification of TRAP-positive multinuclear cells using an acid phosphatase kit (Sigma-Aldrich, Canada), according to the protocols provided by the manufacturer. The bone resorption activity of osteoclasts was tested *in vitro*, using a previously validated model [30]. The details of the TRAP assay and the *in vitro* assessment of bone resorption are provided in the S1 File.

### Quantitative real-time PCR

In order to test if the most strongly PDB associated *OPTN* variant had an impact on its gene expression, we performed qPCR as previously described [12]. The details of this analysis are provided in the S1 File.

### Western blot analysis

Osteoclasts derived from patients and controls PBMCs were washed once in PBS and lysed using Trizol. The protein concentrations were determined using the Bradford reagent (Bio-Rad, Canada). Proteins were separated by 8% SDS-polyacrylamide gels and transferred onto PVDF membranes (Life technologies, Canada). After transfer, the membranes were blocked in 0.1% TBS/Tween 20 containing 5% nonfat dried milk at room temperature for 1 h. The membranes were then incubated overnight at 4°C with anti-OPTN (1:500, #100000 Cayman, Canada), anti-LC3B (1:1000, #2775 Cell Signaling, Canada) or anti-αTubulin (1:2000, #2144 Cell Signaling, Canada) in 0.1% TBS/Tween 20, followed by extensive washing using 0.1% TBS/Tween 20 and an incubation with HRP-conjugated secondary antibody (1: 2000, Cell signaling, Canada) in 0.1% TBS/Tween 20 during 1h, at room temperature. After extensive washing with 0.1% TBS/Tween 20, specific proteins were detected using a chemiluminescence kit (GE Healthcare, Canada). The densitometric analysis was performed using ImageJ software.

### Statistical analysis

Statistical analysis was performed with GraphPad Prism 5 (GraphPad, La Jolla, CA). Comparisons between two groups were made using a two-tailed Student’s t-test. For comparisons between multiple groups, one-way ANOVA followed by Tukey’s post-hoc test was used. Differences were considered statistically significant when p<0.05.

## Results

### Rs1561570 increases *in vitro* osteoclast differentiation and bone resorption activity

**Fig 1:**
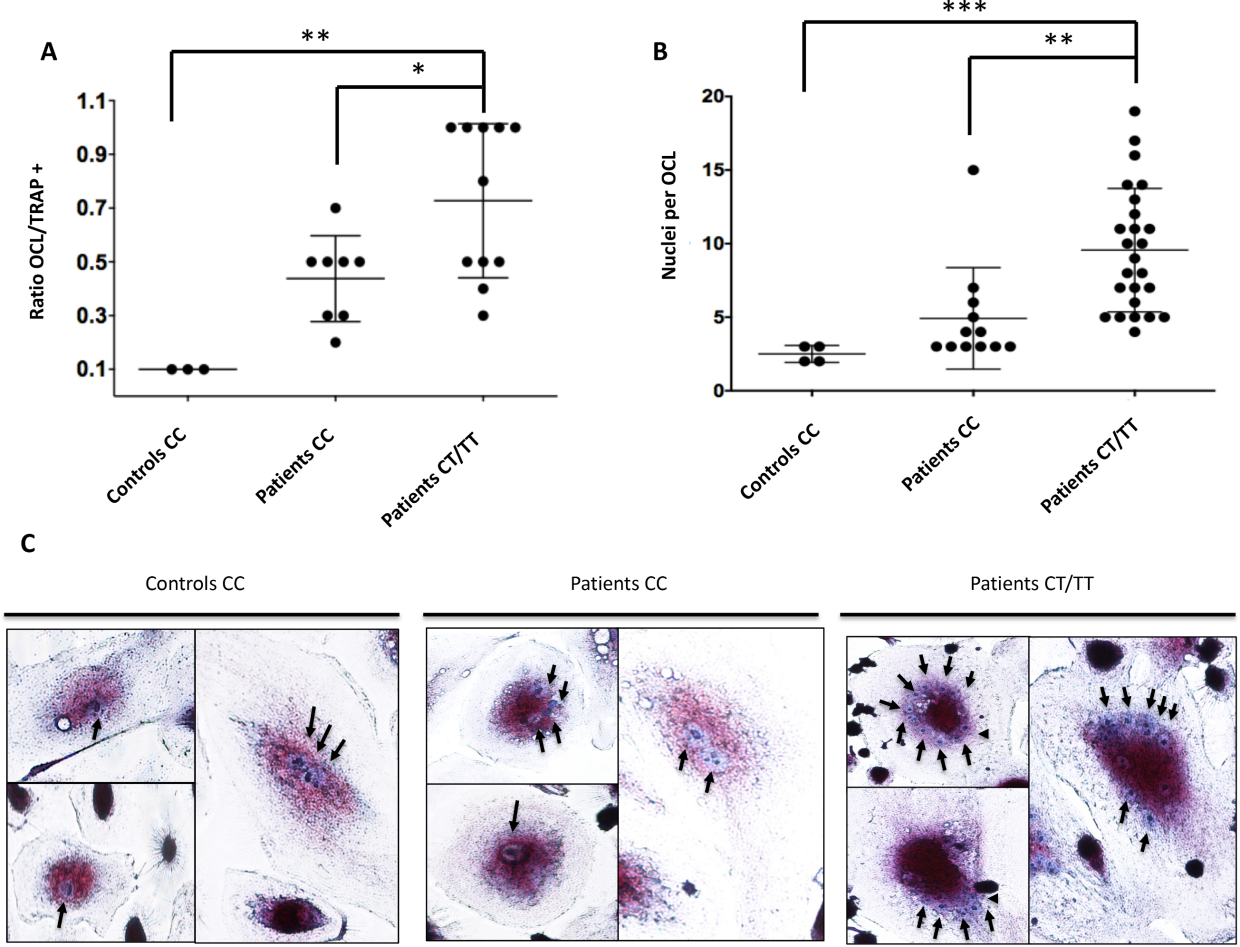
Effect of rs1561570 in osteoclast differentiation. (A) The osteoclast differentiation rate (displayed as a ratio between multinucleated osteoclasts (Multi OCL) and TRAP positive (+) cells) was higher in patients with PDB carrying at least one *T* allele (Patients *CT/TT vs* Controls *CC p*-value < 0.01; Patients *CT/TT vs* Patients *CC p*-value < 0.05). (B) Osteoclasts from patients carrying at least one *T* allele contained significantly more nuclei than osteoclasts from healthy controls (Patients *CT/TT vs* Controls *CC p*-value < 0.001; Patients *CT/TT vs* Patients *CC p*-value < 0.01). (C) The images are representative of the distribution of nuclei number observed in osteoclasts generated from PBMCs derived from healthy controls and PDB patients that were stained for TRAP and counterstained with haematoxylin. (ANOVA, * represents a *p*-value < 0.05, ** represents a *p*-value < 0.01, *** represents a *p*-value < 0.001, **** represents a *p*-value < 0.0001; non-mutated controls with *CC* genotype (n = 3), non-mutated patients with *CC* genotype (n = 5) and patients carrying at least one *T* allele (n = 3).

To better assess the effect of rs1561570 on osteoclasts formation, we performed a TRAP assay in osteoclasts derived from the PBMCs of patients carrying the rs1561570 *C* or the *T* allele. The presence of a haplotype with at least one *T* allele for rs1561570 increased significantly osteoclast formation, attested by the mean number of multinucleated cells yielded from PBMCs cultures, when compared to healthy controls (Controls *CC* 10%, Patients *CC* 50%, Patients *CT/TT* 80%, *p*-value = 0.006) (Fig 1A and 1C). The osteoclasts generated displayed a higher mean number of nuclei than the osteoclasts from healthy controls (Controls *CC* mean = 3, Patients *CC* mean = 5, Patients *CT/TT* mean = 10, *p*-value = 0.002) (Fig 1B and 1C).

**Fig 2:**
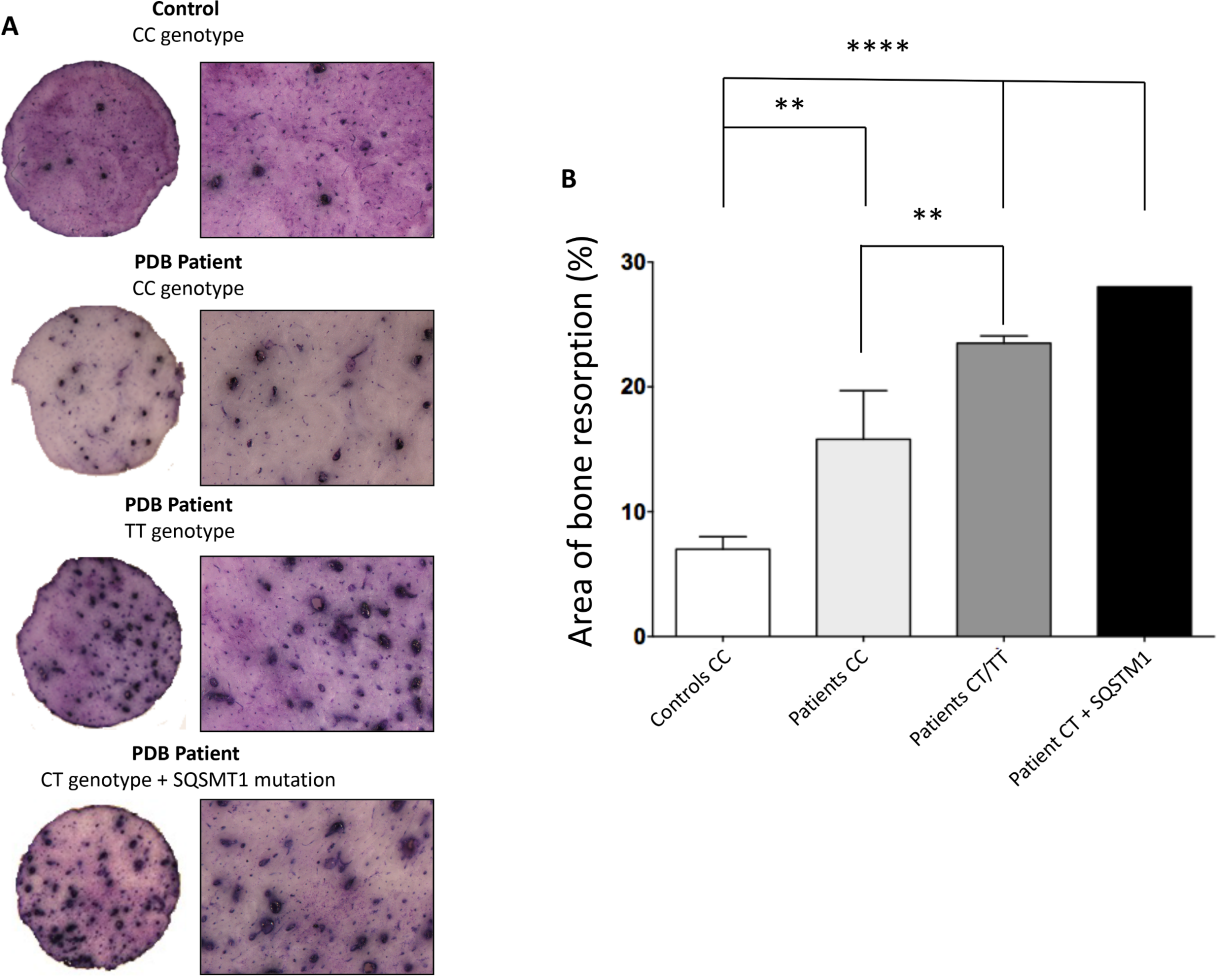
Effect of rs1561570 in bone resorption. (A) Representative images of *in vitro* bone resorption assays (left) with (B) quantification of the results (right) by using ImageJ. The bone resorption area was higher in patients with PDB carrying at least one *T* allele (Patients *CT/TT vs* Controls *CC p*-value < 0.001; Patients *CT/TT vs* Patients *CC p*-value < 0.01). This result was even more evident in one PDB patient carrying one *T* allele (*CT* genotype) plus the *SQSTM1/P392L* mutation (*p*-value < 0.001). At least three different wells per patient were analysed. (ANOVA, * represents a *p*-value < 0.05, ** represents a *p*-value < 0.01, *** represents a *p*-value < 0.001, **** represents a *p*-value < 0.0001; non-mutated controls with *CC* genotype (n = 3), non-mutated patients with *CC* genotype (n = 5) and patients carrying at least one *T* allele (n = 3)).

Accordingly, bone resorption was also significantly increased in osteoclast cultures generated from patients with a haplotype carrying at least one *T* allele for rs1561570, resulting in 3-fold increase in the total bone resorption area when compared to cultures prepared from healthy controls (*p*-value < 0.001) (Fig 2A and 2B) and 1.4-fold increase when compared to cell cultures prepared from PDB patients carrying two *C* alleles (*p*-value = 0.05). We also analysed the bone resorption area from osteoclast cultures of one patient carrying a haplotype with one *T* allele (*CT*) in combination with the *SQSTM1/P392L* mutation. Interestingly, there was an increase of the bone resorption area in the patient with one *T* allele, suggesting that there might be a synergistic effect of *OPTN* and *SQSTM1* genetic variants in PDB (Fig 2B).

### Rs1561570 does not affect autophagy

OPTN has been suggested to be an autophagy adaptor and since impairment in autophagy was reported in PDB, we assessed the autophagy process in patients with a haplotype carrying at least one rs1561570 *T* allele. By immunofluorescence and western blot analysis we found that LC3BII expression was impaired, however that effect did not seem to be dependent of the presence of a haplotype with the *T* allele (S1A and S1B Fig), suggesting that the autophagy impairment may be independent from the rs1561570 genotype.

### Rs1561570 causes a change in methylation and increases *OPTN* expression

Since our previous study has shown a strong association between rs1561570 (located in *OPTN* intron 7) and PDB [12], we determined the functional effect of rs1561570 in that disease by employing the splice site prediction algorithm, and showed that the allele harbouring a *C* was likely to create a potential new acceptor site for splicing and disrupt a potential branch point (consensus value for mutant sequence = 69.7%) (S2 Table). To validate this finding, we performed RT-PCR and qPCR using cDNA samples from patients and healthy controls with the three genotypes. Retention of intronic sequences was not observed in the genotypes analysed (results not shown).

**Fig 3:**
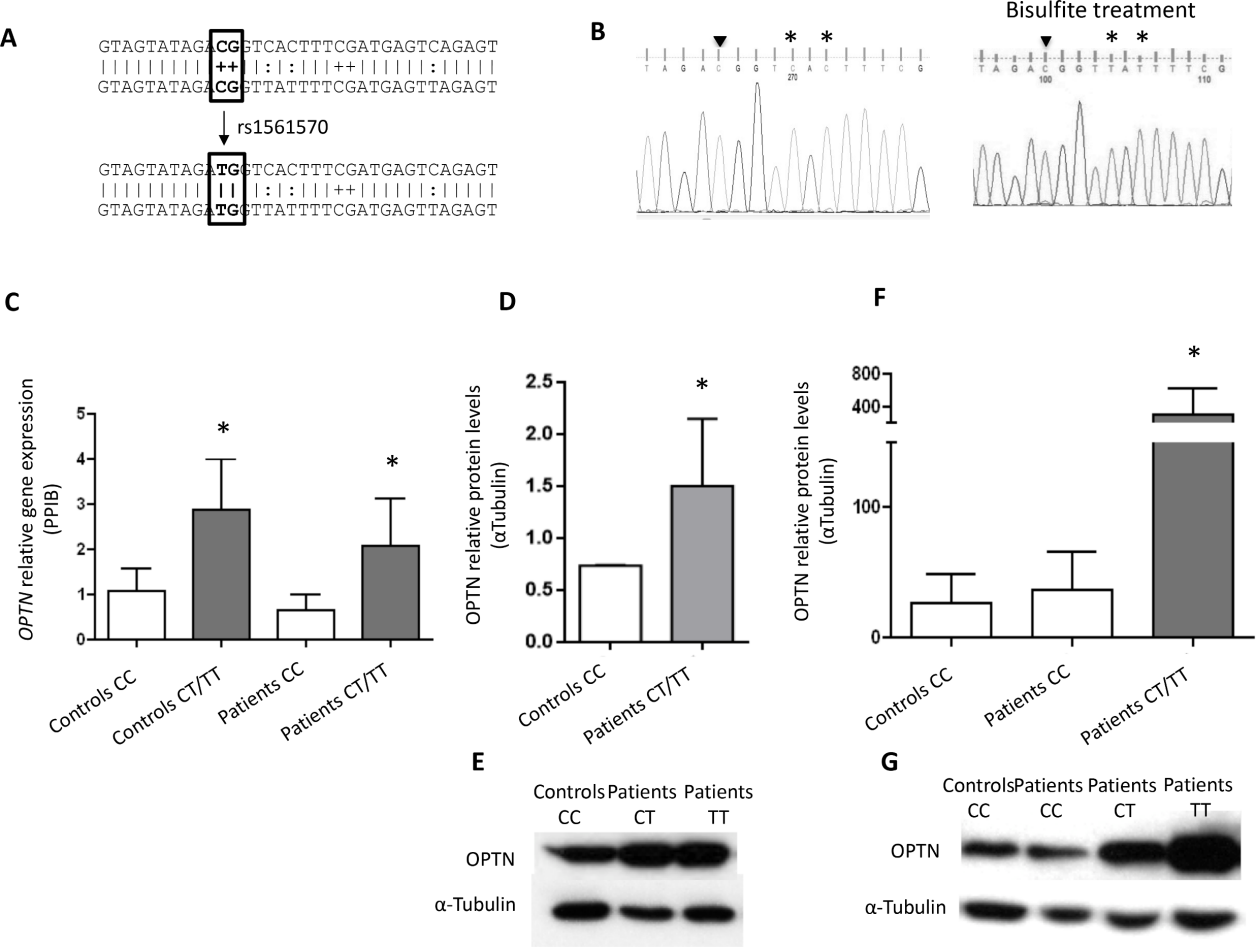
Rs1561570 effect in methylation and *OPTN* expression. (A) *In silico* prediction using Methprimer tool showing a methylation site only in the presence of the allele *C* (not the allele *T*). (B) Sequencing results showing that rs1561570 *C* allele after bisulfite treatment remains a *C* (arrow head), while other *C* nucleotides that are not methylated change to *T* (asterisk). (C) Analysis of *OPTN* gene expression in lymphocytes from several healthy controls (*CC* genotype n = 10, *CT/TT* genotypes n = 6) and PDB patients (*CC* genotype n = 10, *CT/TT* genotypes n = 22) with the three genotypes. The levels of *OPTN* gene expression were measured by qPCR normalized to *PPIB* gene expression levels. Values are the mean of two replicates in each experiment and the experiment was repeated at least three independent times. (D) Quantification and (E) representative blots of OPTN protein expression in lymphocytes from non-mutated controls with *CC* genotype (n = 5), and patients carrying at least one *T* allele (n = 19). (F) Quantification and (G) representative blots of OPTN protein expression in osteoclasts from non-mutated controls with *CC* genotype (n = 3), non-mutated patients with *CC* genotype (n = 5) and patients carrying at least one *T* allele (n = 3). The levels of OPTN protein expression were measured by western blot analysis and related to levels of α-Tubulin (t-test and ANOVA, * represents a *p*-value < 0.05). The figures are representative of all the western blot analyses performed (n = 3).

We then studied the predicted impact of rs1561570 in the *OPTN* methylation status using the Methprimer tool. We found that the allele *C* was predicted to form a CpG dinucleotide, creating a methylation site, which was not predicted in the presence of the *T* allele (Fig 3A). After bisulfite treatment, we confirmed that all samples with at least one *C* allele in their rs1561570 genotype were in fact methylated (Fig 3B). To correlate the methylation site created by the *C* allele with the *OPTN* transcriptional levels, we performed qPCR (Fig 3C) and western blot (Fig 3D) analysis using lymphocytes from patients with the three genotypes. Samples carrying a haplotype with the *T* allele (*CT* or *TT*) showed levels of *OPTN* gene and protein expression higher than samples carrying the *CC* genotype. This result suggested that the methylated *C* allele is associated with a decrease in *OPTN* gene expression levels. The protein expression levels were also measured by western blot analysis in PBMC-derived osteoclasts and the results were in accordance with those obtained by qPCR and western blot analysis in lymphocytes (Fig 3E).

### Rs1561570 increases NF-κB translocation into the nucleus

**Fig 4:**
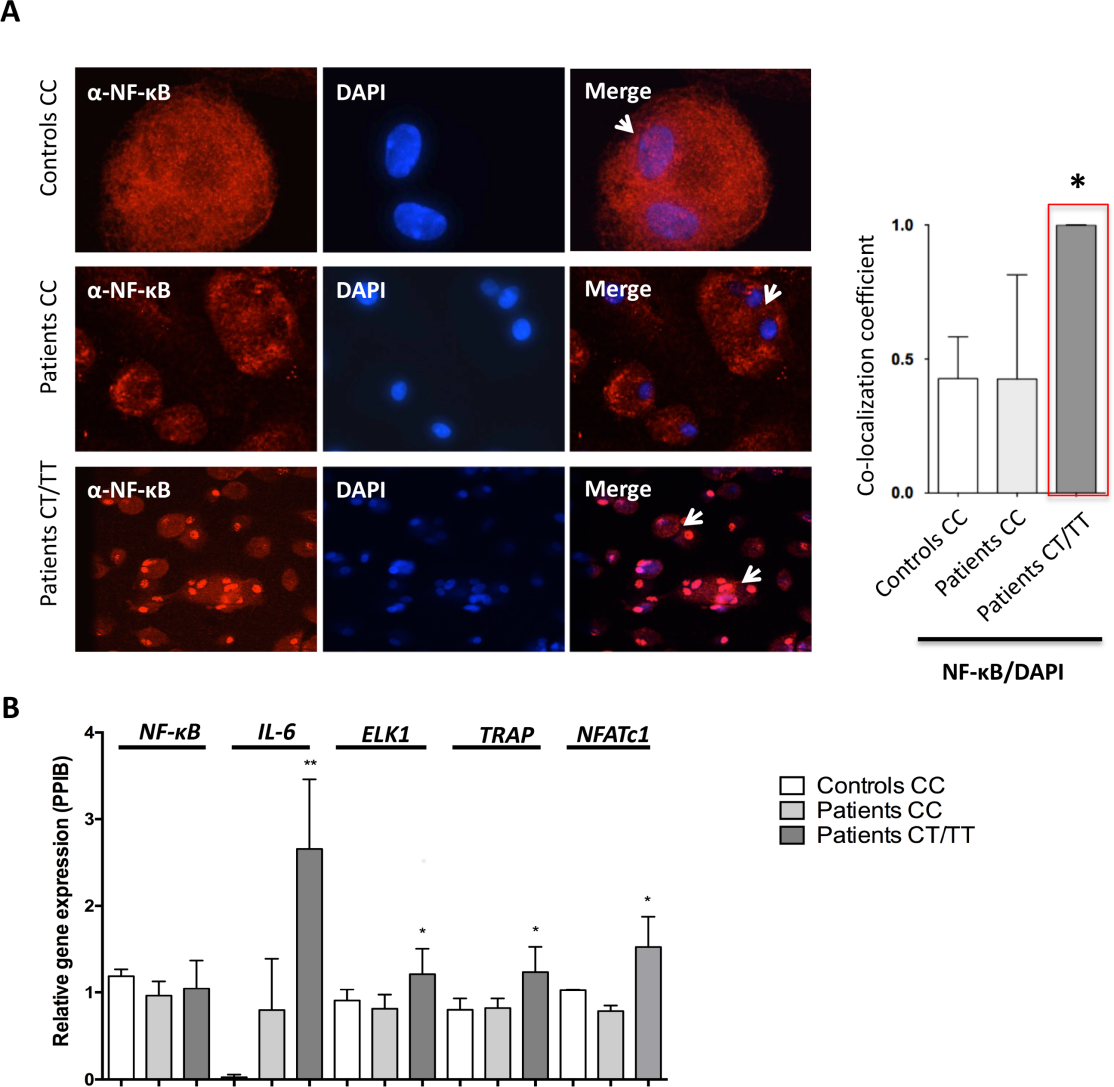
Effect of rs1561570 in NF-κB localization and in the expression of NF-κB target genes in osteoclasts of patients with PDB. (A) NF-κB localization in osteoclasts derived from healthy controls and patients PBCMs showing an increase of the translocation of NF-κB into the nucleus, in patients with genotype *CT* and *TT*. This was also confirmed by co-localization ratio between NF-κB and DAPI–the nuclear staining. Five different fields of view were analysed per sample and at least three different samples were analysed per patient. Co-localization coefficient was calculated by Volocity software as the ratio of NF-κB staining related to DAPI. (B) Analysis of *NFATc1*, *IL6*, *TRAP* and *ELK1* gene expression in several healthy controls (n = 16) and PDB patients (*CC* n = 10, *CT*/*TT* n = 22) with all genotypes. The levels of *NFATc1*, *IL6*, *TRAP* and *ELK1* expression were measured by qPCR related to levels of *PPIB*. Values are the mean of at least three independent replicates. (t-test, * represents a *p*-value < 0.05, ** represents a *p*-value < 0.01).

To determine the role of rs1561570 on the osteoclastic phenotype, and since there are previous publications describing OPTN as an important protein in NF-κB pathway [25], we analysed the NF-κB cellular localization by immunofluorescence in PBMC-derived osteoclasts from PDB patients carrying different genotypes of rs1561570 and from healthy controls. Our results showed NF-κB translocation to the nucleus (showed as a ratio between co-localization of NF-κB and DAPI) is increased in patients with genotype *CT* and *TT* (Fig 4A). To confirm that the presence of NF-κB in the nucleus was affecting the expression of its target genes, we measured the expression of *NF-κB*, *IL-6*, *ELK1*, *TRAP* and *NFATc1* expression. Our results showed an increased expression of NF-κB target genes related with osteoclastogenesis, such as *IL-6*, *ELK1*, *TRAP* and *NFATc1* (Fig 4B)–thus providing additional evidence that an increase of *OPTN* expression observed in the presence of the *T* allele in the *OPTN* methylation status leads to an increase of NF-κB translocation into the nucleus and an increase of its target genes expression.

**Fig 5:**
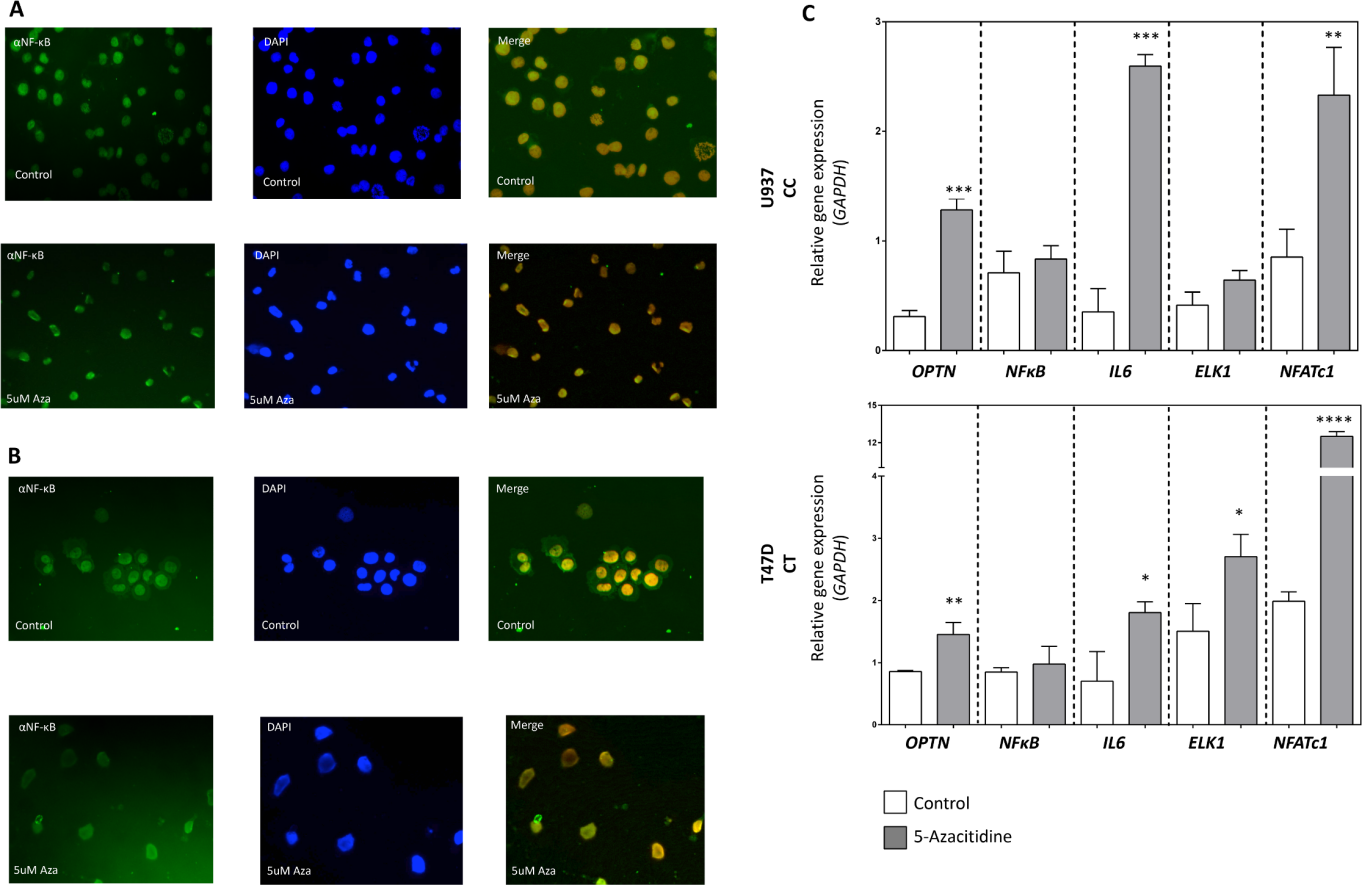
*In vitro* effect of *OPTN* demethylation in NF-κB localization and expression of NF-κB target genes. (A) NF-κB localization in U937 cells (*CC* genotype). (B) NF-κB localization in T47D cells (*CT* genotype), following 5-Azacitidine (5-Aza) treatment. After *OPTN* demethylation, NF-κB was translocated exclusively to the nucleus. (C) Analysis of NF-κB target genes expression. The levels of *OPTN*, *NF-κB* and NF-κB target genes (*IL-6*, *ELK1*, *NFATc1*) expression were measured by qPCR related to levels of *GAPDH* gene. Values are the mean of at least three independent replicates. (t-test, * represents a *p*-value < 0.05, ** represents a *p*-value < 0.01 *** represents a *p*-value < 0.001, **** represents a *p*-value < 0.0001).

As a proof of concept, we also analysed the NF-κB cellular localization by immunofluorescence in two different cell lines which carry the rs1561570 *C* allele–T47D (*CT* genotype) and U937 (*CC* genotype)–following a treatment with a demethylating agent, 5-Azacitidine. We were able to confirm that, before the demethylating treatment, NF-κB was localized in the cytoplasm and in the nucleus. However, after *OPTN* demethylation, NF-κB localization was almost exclusively located in the nucleus (Fig 5A and 5B). To confirm the effect of the demethylating agent we measured *OPTN* expression and to confirm that the presence of NF-κB in the nucleus was also affecting the expression of its target genes (as in patients carrying the *T* allele), we measured the expression of *NF-κB*, *IL-6*, *ELK1*, *TRAP* and *NFATc1*. Our results showed that there was an increase both in *OPTN* expression, which was expected after the demethylation treatment, and in the expression of the NF-κB target genes analysed. NF-κB levels were maintained as expected (Fig 5C).

In addition we performed the same procedure using two osteosarcoma cell lines–U2OS (*CC* genotype) and MG63 (*TT* genotype) and the results showed that the demethylating agent increased OPTN and NF-κB target genes in U2OS while NF-κB expression was maintained (S2A Fig), suggesting that a change in the methylation status due to the effect of the demethylating agent had an effect in OPTN and in NF-κB target genes expression. In contrast, in the MG63 cell line this treatment did not have any effect in the expression of *OPTN* or NF-κB target genes (S2B Fig), suggesting that the effect in *OPTN* expression might be specific for the cells carrying one *C* allele, thus supporting our hypothesis.

### Rs1561570 may have an effect on PDB severity in the presence of *SQSTM1* mutations

By comparing the clinical phenotype of patients with PDB carriers of the *CC* genotype of *OPTN* rs1561570 with patients carrying one or two *T* alleles, we observed a tendency, although not significant, for an: i) increase of total serum ALP (sALP) levels, ii) increase of mean number of affected bones, and iii) increase of disease extension measured by Renier’s index–related with the number of *T* alleles in the genotype (S3 Table). These results suggest that rs1561570 could have a dose effect depending on the number of *T* alleles present in the genotype. In fact, in the presence of the *P392L/SQSTM1* mutation and rs1561570 *T* allele our results showed an increased tendency for i) higher levels of total sALP, ii) higher risk for polyostotic involvement, iii) higher number of affected bones and iv) an increase in the disease extension/Renier’s index (S4 Table) with the increase in the number of *T* alleles. Altogether, these results may suggest a modifier effect of *OPTN* common variant in the presence of *SQSTM1/P392L* mutation in patients with PDB, which could lead to an increase in the disease activity and extent, and therefore should be replicated in independent cohorts with higher number of participants.

## Discussion

In the present study, we investigated the functional role of the most significantly PDB associated genetic variant of *OPTN*- rs1561570 (C>T). Previously, we reported that the *C* allele was more frequent in controls in the French-Canadian population (*C* allele frequency = 52%, *T* allele frequency = 48%) while the *T* allele was more frequent in PDB patients (*C* allele frequency = 36%, *T* allele frequency = 64%) [12]. Using *in silico* tools, we found that the rs1561570 *T* allele was predicted to cause the loss of a methylation site. With bisulfite treatment, we confirmed that the *C* allele in that position was always methylated. Furthermore, both qPCR and western blot analysis correlated the presence of the *C* allele with a decrease of *OPTN* expression levels. The existence of a correlation between the hypermethylation status of a given gene, resulting in a decrease in gene transcription levels affecting a related disease, has been previously reported in the literature [31–33]. Our immunofluorescence analysis also allowed us to confirm that an increase in *OPTN* levels (due to the demethylation treatment in cells and the presence of at least one *T* allele in PDB patients—dominant genetic effect) is related to an increase in NF-κB translocation into the nucleus, confirming that a decrease in *OPTN* methylation (as seen in PDB patients with rs1561570 *T* allele) increases the translocation of NF-κB into the nucleus, and that is related to an increased expression of NF-κB target genes related with osteoclastogenesis, such as *NFATc1* –a master transcription factor of osteoclast differentiation [34], IL6—an important cytokine for osteoclastogenesis [35] and ELK1—a relevant transcription factor in mature osteoclasts [36]. We also showed that the autophagy process is impaired in PDB patients and that it is not dependent on the rs1561570 genetic status. Therefore, we can hypothesize that in healthy individuals, the autophagy process works normally both in the presence of the *C* allele (when the *OPTN* expression is controlled at basal levels), and in the presence of the *T* allele (when the *OPTN* expression is potentiated due to the loss of a methylation site). In the latter, the efficient autophagy process signals the excessive OPTN for degradation by ubiquitination and recycling of the protein, and thus *OPTN* expression is reduced to a basal level. In both situations, this will result in normal levels of NF-κB in the nucleus and physiological osteoclastogenesis rate. In PDB patients, where the autophagy process is impaired, in the presence of the rs1561570 *C* allele, the PDB phenotype might be explained by the presence of other factors, such as other genetic variants or environmental triggers that will lead to an increase in osteoclastogenesis. The effect of rs1561570 *T* allele in PDB patients may therefore be explained by the increase in *OPTN* expression associated with a decrease in its degradation due to the autophagy defect present in these patients. Consequently, there is an accumulation of OPTN that promotes NF-κB translocation to the nucleus at a much higher rate than normal, thus also promoting osteoclastogenesis and that in addition to the existence of other genetic variants or environmental triggers potentiate the clinical phenotype of PDB in these patients.

Journo and its co-workers had already described the translocation of NF-κB into the nucleus due to an increase in *OPTN* expression [25], but upon a virus infection. OPTN is part of a complex, which also includes TAX1 and TAX1BP1, that can activate the IKK complex by ubiquitination, thus leading to the degradation of the NF-κB inhibitor and subsequent release of NF-κB into the nucleus. In the present work, the change in *OPTN* expression levels caused by one single change in nucleotide may be explained by the alteration of the already reported binding of an enhancer—JUND, a member of the AP1 family that is present in osteoclasts [37–39]—in the genomic region containing the *T* allele, which is lost in the presence of the *C* allele, as confirmed by the ENCODE project. The ENCODE project shows regions of transcription factor binding, derived from a large collection of ChIP-seq experiments, together with DNA binding motifs identified within these regions by the ENCODE Factor book repository. Indeed, due to the resulting change in DNA conformation in the presence of the *T* allele, the loss of a JUND binding site around the rs1561570 region of these patients will result in an alteration in *OPTN* expression levels, promoting an increase in osteoclastogenesis and the development of PDB. Our results are in contrast with data from Obaid *et al*. (2015) who, based in the description by Zeller and Westra’s groups (who have shown that rs1561570 *T* allele was related to lower levels of OPTN expression [40,41]), analysed the effect of a lack of OPTN in the differentiation of osteoclasts using a mouse knock out for OPTN. Those authors concluded that depletion of *OPTN* in that model increased the differentiation of osteoclasts (*p*-value = 0.002), suggesting that *OPTN* might be a negative regulator of osteoclast differentiation [24]. However, and, unlike in humans, these mice did not develop a PDB-like phenotype [24]^,^[17]. Interestingly, in the same study, authors also refer that *OPTN* expression was significantly increased during days 2 to 5 of *in vitro* osteoclast differentiation using primary bone-marrow-derived macrophages, in which they knocked down OPTN by using a small hairpin RNA. In addition, it was found in another set of *in vivo* works, using either knock-in mice expressing a point mutation in OPTN and abolishing its polyubiquitin binding capacity [42], or mice lacking either the entire C-terminal ubiquitin-binding [43] or the N-terminal TANK-binding kinase 1 (TBK1)-binding domain [44], that mice had retained normal NF-κB responses. These results argued against the fact that a decrease *per se* in OPTN or in OPTN function could promote osteoclastogenesis by affecting NF-κB signalling pathways.

As referred in a recent review, OPTN was thought to be important for NF-κB signalling because of its strong homology to the NF-κB essential modulator, NEMO—the core element of the inhibitor of NF-κB kinase (IKK) complex -, that is essential for NF-κB activation [18]. Altogether, the lack of a PDB-like phenotype developed in those studies is not in favour of a lack of *OPTN* expression being the causing factor. As suggested in the same review, it is more likely that the PDB phenotype is the result of an aberrant expression of OPTN, including a possible gain of function [18] which is in agreement with our results. Our study is unique since it analyses, using *in vitro* and *ex vivo* techniques, the single effect of rs1561570 in *OPTN* gene and protein expression in several samples of patients with PDB non carriers of a *SQSTM1/P392L* mutation, and in different cell types (lymphocytes and osteoclasts derived from patients PBMCs), thus providing, at several levels, clear evidences supporting our hypothesis. Our finding that, in one patient carrying the *SQSTM1/P392L* mutation, there was a tendency for a more severe clinical phenotype in the presence of a haplotype with the rs1561570 *T* allele and that osteoclasts from that patient had a higher *in vitro* bone resorption rate, suggests that there might be a synergistic effect but this should be further investigated with additional patients.

Altogether, the gathered evidence obtained from gene to protein analysis, from *in vitro* to patient samples, from genetic to morphological cellular features, show that OPTN appears to be important for NF-κB translocation into the nucleus, activation of its target genes and consequent increase in osteoclastogenesis, providing relevant information towards a better understanding on how genetic variants in *OPTN* can contribute to PDB by acting as modifiers and complementing previous data on the potential functional role of variants in OPTN and how they may contribute to affect the physiological regulatory mechanisms of this gene [45].

## Conclusions

In conclusion, we have shown that rs1561570 is responsible for a change in the methylation status of *OPTN* in patients with PDB and that it has an impact in its expression levels, which may contribute to PDB pathogenesis by affecting NF-κB translocation to the nucleus. Accordingly, to this date, *OPTN* remains the most likely gene within PDB6 locus to contribute to PDB pathophysiology, possibly in combination with other genetic/environmental factors yet unknown.

## Supporting information

S1 File Supplementary methods. (DOCX)

S1 Fig rs1561570 effect in autophagy. (a) LC3BII foci (red) in PBMC-derived osteoclasts from controls (with genotype CC, n = 3) and PDB patients (non-mutated patients with CC genotype (n = 5) and patients carrying at least one T allele (n = 3)) were analysed by immunofluorescence. At least three different wells per patient were analysed. (b) The levels of LC3BII protein expression were measured by western blot analysis and related to levels of α-Tubulin (left). Quantification of the results was performed by using ImageJ (right). The figures are representative of all the western blot analyses performed. (ANOVA, * represents a *p*-value < 0.05).
(TIF)

S2 Fig *In vitro* effect of *OPTN* demethylation in expression of NF-κB target genes in U2OS and MG63 cells. The levels of *OPTN*, *NF-κB* and NF-κB target genes (*IL-6*, *ELK1*, *NFATc1*) expression were measured by qPCR related to levels of *GAPDH* gene in (a) U2OS (rs1561570 *CC* genotype) and (b) MG63 cells (rs1561570 *TT* genotype). Values are the mean of at least three independent replicates. (t-test, * represents a *p*-value < 0.05, ** represents a *p*-value < 0.01, *** represents a *p*-value < 0.001, **** represents a *p*-value < 0.0001).
(TIF)

S1 Table Primer list. (XLSX)

S2 Table Splicing effects predicted by human splicing finder. (XLSX)

S3 Table Comparisons of main clinical characteristics between French-Canadian PDB patients carriers of the rs1561570 TT or TC genotype and the CC genotype carriers, considered as the reference. All 232 patients were non-carrier of the P392L mutation within the SQSTM1 gene (PDB3 locus).
(XLSX)

S4 Table Comparisons of main clinical characteristics between French-Canadian PDB patients carriers of the rs1561570 TT or TC genotype and the CC genotype carriers, considered as the reference. All 32 patients were carrier of the P392L mutation within the SQSTM1 gene (PDB3 locus).
(XLSX)
