# Supplementary material for: Molecular effect of an OPTN common variant associated to Paget's disease of bone
Source: PLoS One. 2018 May 21;13(5):e0197543. doi: 10.1371/journal.pone.0197543 (PMC5962077; doi:10.1371/journal.pone.0197543)
Supplement: S1 File — (DOCX) [file pone.0197543.s001.docx]

**Molecular effect of an OPTN common variant associated to Paget’s disease of bone**

Iris AL Silva, ^1, 2#a^ Natércia Conceição, ^1,2^ Édith Gagnon, ^3^ Jacques P Brown, ^3^ M. Leonor Cancela ^1, 2^* and Laëtitia Michou ^3, 4^*

*^1^Department of Biomedical Sciences and Medicine, University of Algarve, Faro, 8005-139, Portugal*

*^2^Centre of Marine Sciences (CCMAR), University of Algarve, Faro, 8005-139, Portugal*

*^3^Research centre of the CHU de Québec-Université Laval, Québec* *City, QC, Canada*

*^4^Division of Rheumatology, Department of Medicine, Université Laval and Department of Rheumatology, CHU de Québec-Université Laval, Québec City, QC, Canada*

* Joint corresponding authors.

#a Present address: Faculty of Sciences, BioISI - Biosystems & Integrative Sciences Institute, University of Lisboa, Campo Grande, 1749-016, Lisbon, Portugal.

*Corresponding authors:

Email: [laetitia.michou@crchudequebec.ulaval.ca](mailto:laetitia.michou@crchudequebec.ulaval.ca) (LM)

Email: [lcancela@ualg.pt](mailto:lcancela@ualg.pt) (MLC)

**SUPPLEMENTARY METHODS**

**Study participants**

This study was approved by the CHU de Québec-Université Laval Ethics Committee and all participants have signed a consent form before inclusion in the study. Phenotype assessment comprised a complete bone evaluation, including total serum alkaline phosphatase measurement, a total body bone scan and skull and pelvis X-rays. We investigated patients with familial form of PDB (one patient per family), unrelated PDB patients and healthy controls, all from the French-Canadian population. Clinical characteristics of these cohorts were previously published [1–3]. For each individual, peripheral blood was obtained by venipuncture and DNA was extracted from blood samples, using standard procedures. All patients and healthy donors studied here were non-carrier of the *P392L* mutation within the *SQSTM1* gene (*PDB3* locus), with the exception of one PDB patient who was carrier of the *T* allele (*CT* genotype) and the *SQSTM1/P392L* mutation and who was studied for the assessment of the osteoclast differentiation. RNA from total blood was collected in a subset of patients and controls, on PAXgene tube (Qiagen) and RNA extraction was performed according to manufacturer recommendations.

**Methylation analysis by bisulfite conversion and Sanger sequencing**

Sodium bisulfite treatment was performed on 1.5 µg of genomic DNA sample using the EpiMark® Bisulfite Conversion Kit (New England Biolabs Inc., Canada) following the manufacturers’ standard protocol. DNA samples from 130 PDB patients and 225 healthy individuals (with *CT* and *CC* genotypes of rs1561570) were bisulfite converted, with fully-methylated (methylation level of 100%) and unmethylated DNA (0% methylation) samples as controls. The samples with incomplete bisulfite conversion were discarded from this analysis. Bisulfite-PCR amplification was conducted using Taq DNA polymerase and cycling conditions of 45 cycles with an annealing temperature of 56°C. The primers used (Rs1561570 Bisulfite F and Rs1561570 Bisulfite R; Table S1) amplify a region of 267 bp around the rs1561570 genomic position. Methylated cytosine was detected by Sanger sequencing of the PCR products.

**Demethylating treatment and immunofluorescence**

The localization of NF-κB in T47D and U937 cell lines treated for 72h with 5-Azacitidine (5-Aza) and in osteoclasts derived from Peripheral Blood Mononuclear Cells (PBMCs) from controls and patients, was assessed by immunofluorescence. The cells were washed with phosphate buffered saline (PBS), fixed with 4% paraformaldehyde, and permeabilized in PBS containing 0.1% Triton 100 for 4 min. Non-specific binding was blocked by incubating the cells with PBS supplemented with 0.5% FBS and 0.5% bovine serum albumin (BSA) for 30 min at room temperature. Cells were then incubated overnight (ON) at 4^o^C with a rabbit polyclonal antibody directed against human NF-κB (#8242, Cell Signaling, Portugal) or against LC3B (#2775 Cell Signaling, Canada). After rinsing with PBS, the cells were incubated with the secondary antibody Alexa 488 (green) or Alexa 594 (red) goat anti-rabbit (Cell signaling, Portugal), in 0.5% BSA-PBS for 45 min. The microscope slides were rinsed and mounted with the mounting medium for fluorescence.

**Osteoclast morphology assessment and TRAP assay**

Mature osteoclast formation was evaluated by quantification of TRAP-positive multinuclear cells using an Acid Phosphatase kit (Sigma-Aldrich, Canada), according to the manufacture. Briefly, after fixation, the cells were stained for acid phosphatase in the presence of 0.05 M sodium tartrate. The substrate used was napthol AS-BI phosphate. TRAP-positive and TRAP-negative cells were counted in at least five random fields using light microscopy.

***In vitro* assessment of bone resorption**

The bone resorption activity of osteoclasts was tested *in vitro*, using a previously validated model [4]. Briefly, monocytes were seeded onto cortical bone slices and differentiated as described previously. At the end of the culture, cells were removed and bone slices were stained with toluidine blue to score the percentage of bone resorbed area. Bone resorption was evaluated using ImageJ by doing the ratio of the total bone resorbed area versus the total bone slice area.

**Quantitative real-time PCR**

In order to test if the most strongly associated SNP with PDB in *OPTN* had an impact on its gene expression and to analyse NF-κB target genes expression, we performed qPCR as previously described [5]. For this analysis we used RNA from PBMCs of 32 patients and 16 healthy donors. Normalization was performed using the reference gene peptidylprolyl isomerase B (*PPIB*), since it was defined as a suitable reference gene for mRNA quantiﬁcation in peripheral whole blood [6]. To test the gene expression of *OPTN* and NF-κB target genes (*NF-κB, IL-6, ELK1,* and *NFATc1*) in U2OS, MG63, U937 and T47D cells, normalization was performed using the reference gene glyceraldehyde 3-phosphate dehydrogenase (*GAPDH*), since it was defined as a suitable internal control to evaluate gene expression after treatment with 5-Azacitidine [7,8]. A quantity of cDNA corresponding to 10 ng of total RNA was used in these analyses. The primers used are displayed in S1 Table.

**REFERENCES**

1. Morissette J, Laurin N, Brown JP. Sequestosome 1: mutation frequencies, haplotypes, and phenotypes in familial Paget’s disease of bone. J Bone Miner Res. 2006;21 Suppl 2: P38-44. doi:10.1359/jbmr.06s207

2. Laurin N, Brown JP, Morissette J, Raymond V. Recurrent mutation of the gene encoding sequestosome 1 (SQSTM1/p62) in Paget disease of bone. Am J Hum Genet. 2002;70: 1582–8. doi:10.1086/340731

3. Michou L, Morissette J, Gagnon ER, Marquis A, Dellabadia M, Brown JP, et al. Novel SQSTM1 mutations in patients with Paget’s disease of bone in an unrelated multiethnic American population. Bone. Elsevier Inc.; 2011;48: 456–60. doi:10.1016/j.bone.2010.11.004

4. Itzstein C, Espinosa L, Delmas PD, Chenu C. Specific antagonists of NMDA receptors prevent osteoclast sealing zone formation required for bone resorption. Biochem Biophys Res Commun. 2000;268: 201–209. doi:10.1006/bbrc.2000.2097

5. Michou L, Conceição N, Morissette J, Gagnon E, Miltenberger-Miltenyi G, Siris ES, et al. Genetic association study of UCMA/GRP and OPTN genes (PDB6 locus) with Paget’s disease of bone. Bone. Elsevier Inc.; 2012;51: 720–8. doi:10.1016/j.bone.2012.06.028

6. Pachot A, Blond J-L, Mougin B, Miossec P. Peptidylpropyl isomerase B (PPIB): a suitable reference gene for mRNA quantification in peripheral whole blood. J Biotechnol. 2004;114: 121–4. doi:10.1016/j.jbiotec.2004.07.001

7. Kobune M, Iyama S, Kikuchi S, Horiguchi H, Sato T, Murase K, et al. Stromal cells expressing hedgehog-interacting protein regulate the proliferation of myeloid neoplasms. Blood Cancer Journal. 2012. p. e87. doi:10.1038/bcj.2012.36

8. Wang R, Löhr C V., Fischer K, Dashwood WM, Greenwood JA, Ho E, et al. Epigenetic inactivation of endothelin-2 and endothelin-3 in colon cancer. Int J Cancer. 2013;132: 1004–1012. doi:10.1002/ijc.27762
