# Supplementary figures and images for: Molecular effect of an OPTN common variant associated to Paget's disease of bone
Source: PLoS One. 2018 May 21;13(5):e0197543. doi: 10.1371/journal.pone.0197543 (PMC5962077; doi:10.1371/journal.pone.0197543)

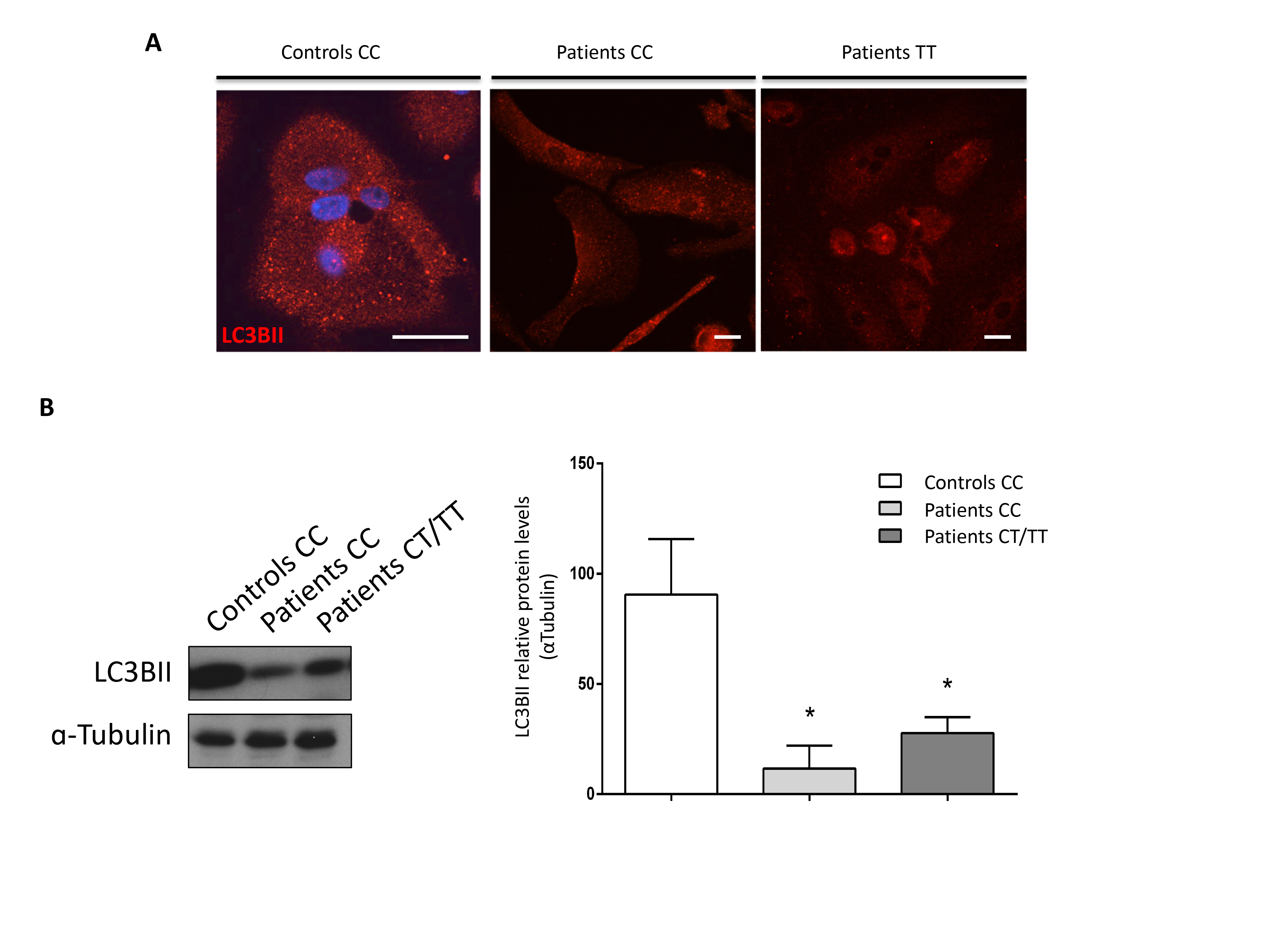

Supplement: S1 Fig — (a) LC3BII foci (red) in PBMC-derived osteoclasts from controls (with genotype CC, n = 3) and PDB patients (non-mutated patients with CC genotype (n = 5) and patients carrying at least one T allele (n = 3)) were analysed by immunofluorescence. At least three different wells per patient were analysed. (b) The levels of LC3BII protein expression were measured by western blot analysis and related to levels of α-Tubulin (left). Quantification of the results was performed by using ImageJ (right). The figures are representative of all the western blot analyses performed. (ANOVA, * represents a p-value < 0.05). (TIF) [file pone.0197543.s002.tif]

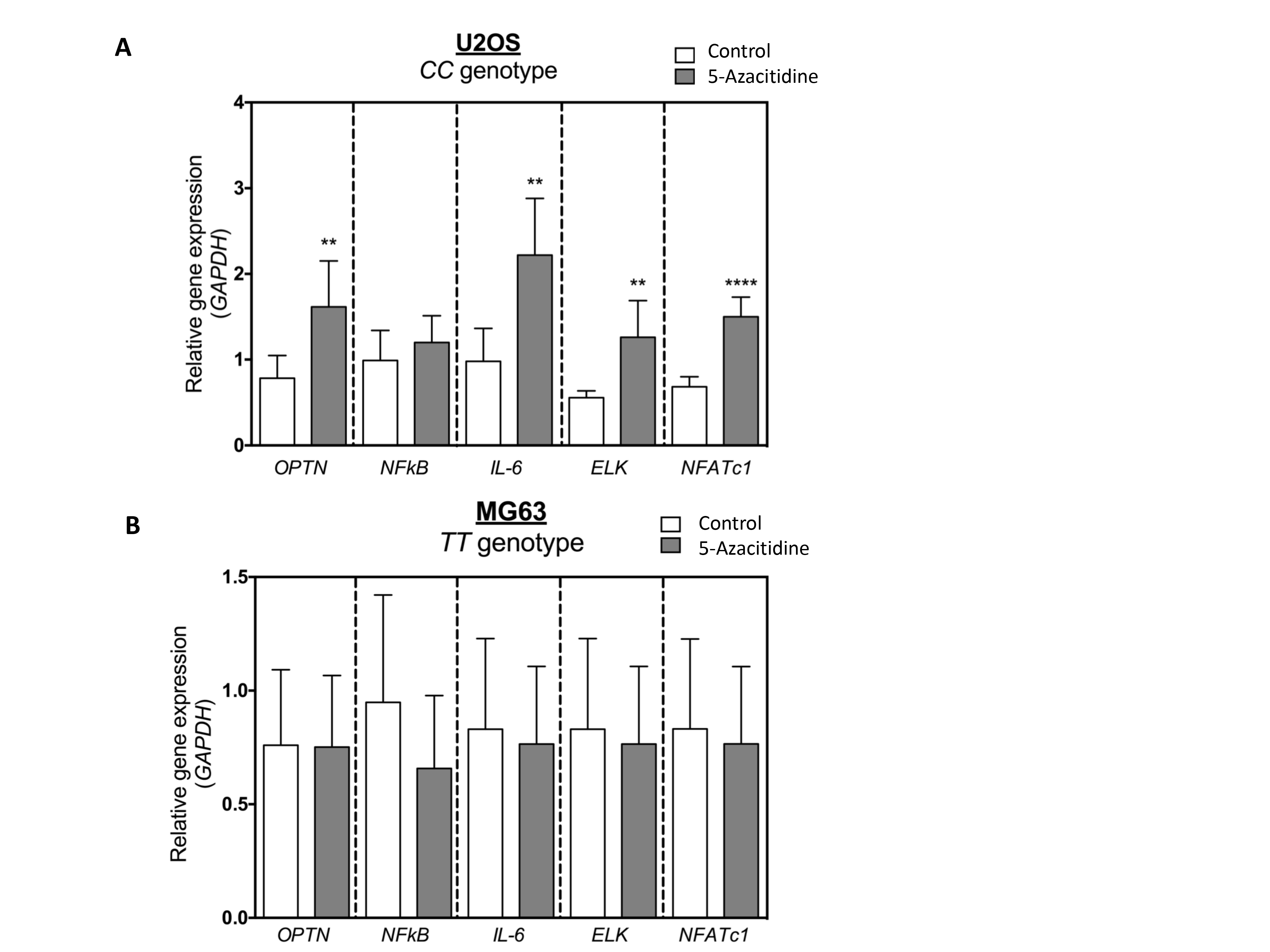

Supplement: S2 Fig — The levels of OPTN, NF-κB and NF-κB target genes (IL-6, ELK1, NFATc1) expression were measured by qPCR related to levels of GAPDH gene in (a) U2OS (rs1561570 CC genotype) and (b) MG63 cells (rs1561570 TT genotype). Values are the mean of at least three independent replicates. (t-test, * represents a p-value < 0.05, ** represents a p-value < 0.01, *** represents a p-value < 0.001, **** represents a p-value < 0.0001). (TIF) [file pone.0197543.s003.tif]
